# Dr. Frank E. L. Brecht

**Published:** 1918-12

**Authors:** 


**Dr. Frank E. L. Brecht,** Buffalo 1872, one of the oldest practicing physicians of Buffalo, died at his home Oct. 30, of influenza, aged 73. He was born in Saxony but had lived in Buffalo for 60 years. He was assistant health physician in the early 70's under the late Dr. E. C. W. O'Brien.

